# Attention unleashed: Creative therapy for thoughtful transformation

**DOI:** 10.1016/j.isci.2026.115387

**Published:** 2026-03-27

**Authors:** Sarah Bou Sader Nehme, Emiliano Macaluso, Radwa Khalil

**Affiliations:** 1 University of Bordeaux, CNRS, Institute of Neurodegenerative Diseases, IMN, UMR 5293, Bordeaux, France; 2 Department of Biology, Faculty of Arts and Sciences, Holy Spirit University of Kaslik, Jounieh, Lebanon; 3 Lyon Neuroscience Research Center, University of Lyon 1, INSERM U1028, CNRS UMR5292, Lyon, France; 4 School of Business, Social, and Decision Sciences, Constructor University, Bremen, Germany; 5 Sorbonne Université, INSERM, CNRS, Institut de la Vision, 75012 Paris, France

**Keywords:** neuroscience, cognitive neuroscience, psychology

## Abstract

Various factors—motivation, interest, fatigue, and external stimuli—influence creative mental processes and attention control (AC). Creative thinking (CT) relies on AC and involves coordinated neural networks and pathways. The cognitive function of AC requires the capacity to direct attention toward distinct features of the environment or internal thoughts. Such selectivity is a key to limiting distractions, preserving focus, and assimilating the essential information needed for CT. Despite progress in creativity research, characterization of underlying neural mechanisms remains limited. To address this gap, we identify the obstacles slowing progress in this area and suggest strategies to overcome them. Moving forward, establishing a collaborative research agenda across multiple disciplines is central to continuing the progress made to date. By fostering such an interdisciplinary research agenda can open new therapeutic avenues for attention-deficit/hyperactivity disorder.

## Graphical abstract

**Figure:**
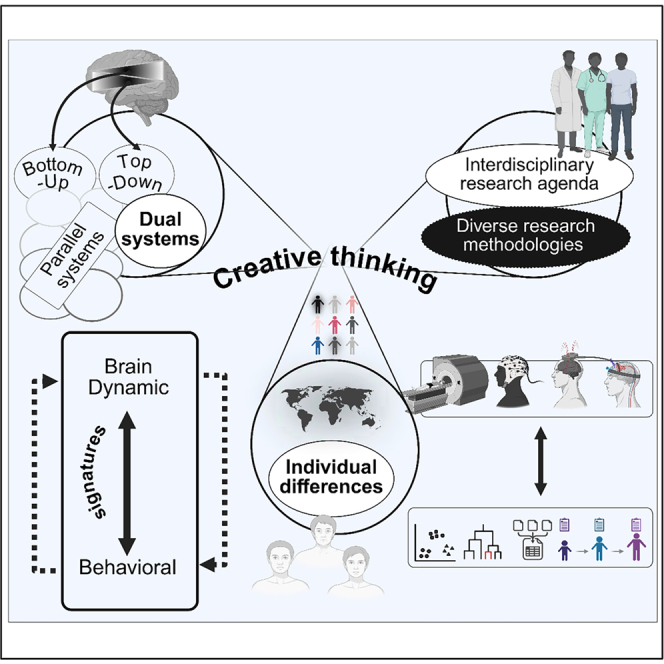

## Introduction

Creative thinking (CT) involves generating novel solutions by recombining existing ideas. It depends on a range of cognitive processes, which makes interindividual variability essential.^1^^,^^2^^,^^3^^,^^4^^,^^5^^,^^6^^,^^7^ Personality traits and motivation also influence CT through these individual differences.^1^^,^^2^^,^^3^^,^^4^^,^^5^^,^^6^ A multitude of cognitive processes contribute to CT: inhibition (i.e., cognitive, attentional, and latent disinhibitions), attention (i.e., diffuse and defocused attention), thinking (i.e., overinclusive and lateral thinking), and memory.^8^^,^^9^^,^^10^^,^^11^^,^^12^^,^^13^ Although cognitive theories based on these constructs do not constitute a unified theory of creative ideation in the traditional sense, they offer a reasonable framework for explaining CT, phenomenologically and neurophysiologically.^14^^,^^15^

From a phenomenological standpoint, Mendelsohn and Griswold were pioneering theorists who explained the unique variations in CT among individuals.^16^^,^^17^ Their formulation emphasized the retrieval of information from conceptual networks in CT and the regulation of attention control (AC). They underscored the effects of operating working memory (WM) and associative processing by evaluating how low cognitive load influences the remoteness of retrieved associations.^16^^,^^17^ The quantity of associative items in the attentional stream limits the number of their associations when they are combined.^16^^,^^18^ Therefore, adding more items to this stream increases the number of combinations. As a result, these individuals can generate unusual ideas by combining a broader range of resources within their attentional framework. Mendelson and Griswold explained these differences in the context of conceptual knowledge, highlighting the value of accessing or retrieving it.^16^^,^^17^

This notion has profound roots in the psychoanalytic tradition, dating back to the 1950s. At that time, Schnier and Kris proposed that CT emerges effortlessly through cognitive transitions between primary and secondary processes.^19^ The primary process involves free-associative (forming connections without logical order) and analogical (drawing parallels between ideas) thinking.^20^ This process often occurs during periods of distractibility, such as fantasy, reverie (daydreaming), and dreaming.^9^^,^^21^ In contrast, the secondary process embodies abstract and logical reasoning, anchored in conscious reality.^15^ Within this framework, CT develops from a regression to the primary process state.^19^ This regression facilitates broader associative thinking, enabling the secondary process to generate novel combinations of these elements. Building on this, Mendelson and colleagues proposed that biased information processing, characterized by poor AC or increased distractibility, may enhance CT.^22^^,^^23^ There is a distinction in the role of AC in accessing knowledge between highly creative individuals and those with lower CT abilities.^7^^,^^8^^,^^18^^,^^24^ Specifically, the attentional stream functions as a spotlight, focusing on information retrieval from memory.^8^^,^^24^ When the spotlight is narrower, it diminishes the stream's focus and impedes the retrieval of conceptual elements.^8^^,^^24^ This process benefits goal-directed action by enhancing the processing of task-relevant information and reducing distractions.^25^ Recently, three views have summarized the explanations for CT variability within the context of AC. They are: (1) internal directed attention (attention focused inward on thoughts or memories),^18^^,^^24^^,^^26^^,^^27^^,^^28^ (2) speed of information processing and levels of arousal,^28^^,^^29^^,^^30^^,^^31^^,^^32^ and (3) broader associative hierarchies in stored conceptual knowledge.^33^^,^^34^^,^^35^^,^^36^

In contrast to phenomenological evaluation, the neurophysiological (brain-based) mechanisms underlying CT via AC remain elusive. This gap stems in part from the separation between psychology and neuroscience. To address this issue, we propose integrating contemporary perspectives on the neural basis of CT through the lens of AC. On this basis, we advocate for promoting interdisciplinary dialogue to overcome current limitations. The terms "creative therapy," "creative therapies," "creative art therapy," and "creative art therapies" refer to approaches using creative expression—such as art, music, movement, and drama—to enhance mental and emotional well-being.^37^^,^^38^^,^^39^ We gave these terms an acronym: CATs. In this context, CATs serve as umbrella terms for interventions involving multiple art forms for therapeutic purposes. We discuss possibilities for advancing the research agenda to benefit individuals with neurodevelopmental disorders characterized by attention deficits, such as attention-deficit/hyperactivity disorder (ADHD). Characterizing the neural mechanisms underlying creativity in ADHD facilitates more precise therapeutic evaluation. ADHD is a neurodevelopmental disorder that ranks among the most prevalent in childhood. It impacts 7.6% of children and 5.6% of adolescents globally.^40^ It may persist into adulthood, with an estimated prevalence of 3.1%.^41^ The etiology and neurobiological basis of ADHD are complex,^42^ involving both genetic and environmental factors that contribute to its development.^43^

In the following sections, we underline what surpasses AC within the framework of CT and outline the neurophysiological mechanisms involved. Subsequently, we provide a brief overview of CATs for ADHD, discuss the associated challenges, and offer recommendations, culminating in a conclusion.

## Beyond attention control

**Figure 1:**
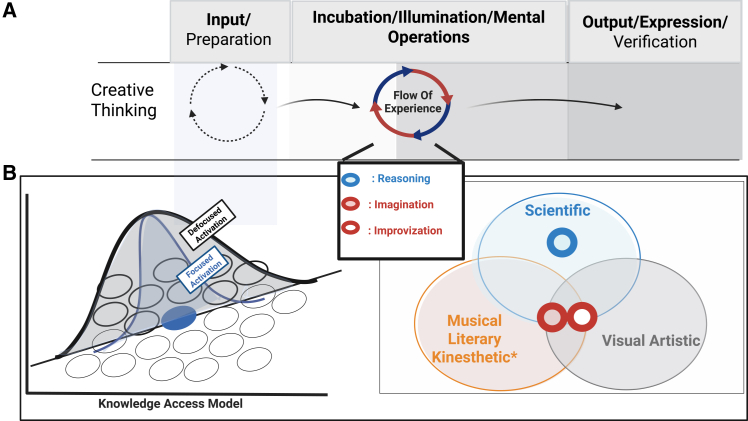
Stages of creative thinking based on the knowledge access model and the potential overlap among various domains of creativity (A) illustrates the three stages of creativity: input or preparation, mental operations encompassing incubation and illumination, and output or expression. The input stage is common to all domains of creativity. The conventional view of creativity posits a sequence of mental operations, followed by the incubation and illumination stages of idea generation, which may vary across domains. The defining feature of this phase could be the experience of flow, which manifests differently across creative domains, including scientific, visual, artistic, musical, literary, and kinesthetic expressions. The star symbolizes the unique emphasis of the kinesthetic domain on perceptual and sensory experiences, differentiating it from other types of creativity. This experience distinguishes various domains of creativity based on the constructs they represent: reasoning, imagination, and improvisation. The extent to which they intersect determines the domains of creativity, such as scientific, musical, literary, kinesthetic, and visual artistic creativity. (B) refers to Mendelson's (1976) knowledge access model. It elucidates the semantic hierarchy by activating defocused and focused attention. Experiential processes modulate the interaction between these two attentional pathways.

Creativity is multifaceted. It can be expressed in a variety of forms, colors, and shapes, which makes it difficult to define.^44^^,^^45^^,^^46^^,^^47^^,^^48^ Although creativity spans several categories—musical, literary, visual, kinesthetic, and scientific—these all share three common stages: input or preparation, mental operations such as incubation and illumination, and output or expression,^49^^,^^50^^,^^51^^,^^52^ as illustrated in Figure 1.

Following this broad framework, Graham Wallas proposed a four-stage theory of the creative process describing how original ideas form.^53^ These stages are preparation, incubation, illumination, and verification (Figure 1A). The first three stages involve unconscious thought. Multiple levels of empirical interaction characterize each of these stages. A key challenge in the preparation phase is balancing existing knowledge with novel ideas.^54^ The incubation phase has been extensively studied, but the findings are mixed. Some research suggests that it positively influences creative problem-solving,^55^ while other studies indicate no significant benefits.^56^ Certain factors require more in-depth analysis, such as the mental effort involved during the incubation phase and the time delays in this phase.^52^^,^^57^ For example, tasks that require low cognitive demand show more pronounced incubation effects than rest periods. In contrast, tasks with high cognitive demands exhibit reduced incubation effects, indicating an inverted-U pattern. A short incubation period is more effective for creative problem-solving than a prolonged one. However, many unresolved questions persist (Box 1).

Box 1 **Outstanding questions**

• What is the optimal level of prior knowledge for creativity? We can approach this from both a tension and a foundation perspective. Is there a threshold of prior knowledge that maximizes creativity but avoids rigidity? This raises the question of how prior knowledge and creativity interact across contexts. When examining CT from the perspective of AC, questions remain about the process's stages. For example, how do focused and defocused attentional activations manifest at different CT stages? Can they coexist within a single stage?
• Given the varied findings about the functional role of incubation in CT, under what circumstances do incubation periods lead to higher creative outcomes? When do they not? Should we consider factors during the incubation period, such as the task's cognitive demands or the length of the delay? For example, do demanding tasks show minimal incubation effects? Do tasks with less cognitive load show stronger effects compared to rest? Finally, does immediate incubation give more benefit for CT than delayed incubation?
• There is a limited number of animal models for studying creativity, with research primarily based on humans. However, these existing models fall short of clarifying the neural mechanisms underlying CT. One of the main reasons for this discrepancy is that creativity manifests differently in humans and animals. While studies of creativity in rodents might not fully capture the nature of conceptually recombinative creativity, they nonetheless raise a vital question: how closely can their creative and innovative behaviors be equated with human creativity?
• Clarifying how CATs' mechanisms influence long-term neural change and the roles of flow and comfort in sustaining focus and CT benefits individuals with ADHD. These efforts enable these individuals to leverage their creative strengths. However, it remains unclear how long CATs induce lasting changes in neural circuits associated with AC. Is there an appropriate/optimal level of distraction removal to support AC during behavioral emotion regulation therapies? To what extent does immersion in the creative process—characterized by flow, a deep state of focus, and intrinsic motivation—affect CATs in relation to AC? How do social and environmental factors interact with behavioral and neural factors to improve personalized interventions and support the preservation of creative strengths in individuals with ADHD?

The illumination phase is the most extensively studied; conversely, the verification phase is the least examined because it is assumed that the cognitive processes involved are nearly identical to those in non-creative tasks. However, some analyses reveal that a thorough examination of Wallas’s concept^53^ identifies five phases, suggesting that an initial intuition phase precedes the illumination phase. These two stages, in turn, mirror dissimilar forms of awareness associated with the creation of insightful ideas: fringe consciousness and focal consciousness.^58^ Despite these perspectives, critiques of the Wallas model^53^ often exhibit analogous deficiencies. One of the most contentious issues in Wallas’ model is whether there is a clear distinction between conscious and unconscious processing across all phases (Box 1).

Building upon these phases, a typical melody across these conceptualizations is characterized by the weight of loosened associational thinking, which arises from various biases in information processing (Figure 1B). The first two concepts—flat associative hierarchies and defocused attention—are grounded in the representation and retrieval of conceptual knowledge (Figure 1B). Therefore, this process engages brain regions within the semantic neural network. Mednick’s flat associative hierarchy model proposed the structural organization of long-term memory networks.^59^ Therefore, this process engages brain regions within the semantic neural network, which organizes long-term semantic memory for knowledge retrieval, as described in Mendelsohn's model of defocused attention^16^^,^^17^ The reason for discussing Mendelsohn’s is that it represents one of the earliest formulations emphasizing the value of retrieving information from conceptual networks associated with CT. Mendelsohn and Griswold also highlight the dual functional roles of AC and WM: adaptable manipulation of representations occurs within the attentional stream.^16^^,^^17^

Expanding on this model, variations in cognitive load or WM demands are associated with the proximity of the retrieved associations.^60^ Increased cognitive load adversely impacts the capacity to retrieve remote associations by narrowing AC. In contrast, when cognitive load is low, activating broader associations automatically triggers an exploratory process. These findings have noteworthy implications for the interaction between knowledge-access-based and knowledge-organization-based frameworks in creative ideation. Nevertheless, several questions remain regarding whether the different functions of AC operate simultaneously during the phases of the Wallas model^53^ (Box 1).

Continuing with the interplay between attention systems, crosstalk between focused and defocused attention pathways shapes the experience of flow.^61^^,^^62^ According to Csikszentmihalyi,^63^ flow is “an almost automatic, effortless, yet highly focused state of consciousness.” It is associated with peak performance across various creative activities, including writing, music, visual arts, and performing arts.^49^ The transient hypofrontality theory^49^ serves as the leading theoretical framework for interpreting the flow experience. Implicit and unconscious information processing, along with the brain systems that support them, are emphasized in facilitating flow.^49^ This phenomenon occurs when the frontal brain temporarily diminishes its executive or cognitive control over other cognitive and neurological processes. While behavioral evidence supports the connection between flow and creative performance, the hypothesis that transient hypofrontality^49^ underlies or accelerates flow states has not yet been empirically validated.

The experience of flow in various activities requires a balance between challenge and skill, a merging of action and awareness, sharp objectives, immediate feedback, intense concentration, control over the situation, reduced self-awareness, an altered perception of time, and intrinsic satisfaction.^49^^,^^62^^,^^64^ The first three elements directly impact the flow experience, while the last six constitute the flow state itself. Flow states are frequently examined in relation to musical performance, encompassing both instrumental play and vocalization.^65^ This state occurs when specific interactions between internal and external factors align during sensorimotor task performance.^64^^,^^66^^,^^67^ Profound engagement in this task should accompany increasing motivation and enthusiasm throughout its execution, leading to a state of flow. However, this task should be suitably challenging, match one’s current abilities, and provide rapid feedback throughout the process. One key feature of this phenomenon is the lack of distinct awareness of time during the “peak experience.”^49^

Building on these features of the flow state, there is a profound sense of joy in fully immersing oneself in the present moment.^49^ The degree to which individuals experience flow is positively associated with personality characteristics such as novelty-seeking, persistence, and self-transcendence.^68^ In contrast, self-directedness negatively correlates with these experiences.^68^ Previous empirical evidence underlines the significance of perceptual and cognitive inputs in the flow experience, and their influence on motivational persistence and positive affect.^64^^,^^67^ Extended practice durations increased the likelihood of attaining flow during musical performances among vocalists, regardless of musical genre.^69^ The self-reported flow experienced by professional classical pianists during the performance of a musical composition showed substantial correlations with cardiovascular and respiratory system metrics, as well as electromyographic activity.^70^ The sense of flow was associated with increased activation, accompanied by deep breathing and engagement of the zygomaticus major muscle, also known as the "smile muscle."^70^ Remarkably, rehearsal duration and emotional intelligence predict the probability of experiencing flow during a musical performance.^71^

Transitioning from behavioral and physiological aspects to the neural underpinnings, the semantic and episodic memory systems are crucial reservoirs that drive idea generation.^72^^,^^73^^,^^74^^,^^75^ One of the well-established hypotheses in this context is the constructive episodic simulation hypothesis.^76^ This hypothesis posits that engaging in episodic and autobiographical recollection can strengthen subsequent associative processes, thereby promoting the development of novel creative ideas.^75^ Traditional theories of episodic memory emphasize the functional roles of the medial temporal cortex (MTC) and ventromedial part of the prefrontal cortex (PFC) in supporting associative processes that connect items to their contexts. This association includes the binding model of items and contexts^77^ and schema-mediated memory.^78^ The potential role of the MTC in CT stems from its involvement in flexible cognition^79^ and its contributions to imagination^9^ and episodic memory.^73^ Schema-based memory^78^ combines prior experiences with current contextual information, serving as a flexible and adaptive mechanism in CT. The ventromedial part of the PFC is associated with the posterior medial memory system.^80^ This memory system, involved in episodic retrieval, represents rich spatiotemporal contexts and integrates prior knowledge of everyday situations.^81^^,^^82^^,^^83^ The posterior medial memory system encompasses the posterior medial parietal cortex (MPC), which is associated with broader aspects of self-generated processing, such as visual imagery^84^ as well as mechanisms involved in reconstructing memory during retrieval.^85^ This system, which includes the MPC, ventromedial PFC, and MTC, largely overlaps with the default mode network (DMN). This overlap may serve as a substrate for integrating pre-existing episodic knowledge to form novel, unique associations during CT.^12^^,^^86^ Episodic induction exhibits further extensive activation in the MPC, the ventromedial part of PFC, and the lateral inferior parietal cortex than semantic induction.^74^ Furthermore, subsequent multivariate decoding analyses of the same dataset revealed that the MPC is involved in semantic integration.

## Neurophysiological mechanisms underlying creativity and attention

Beginning in the 1970s, Martindale pursued unraveling the neural correlates of creativity by conducting an EEG to examine brain activity elicited during CT. Martindale and colleagues reported that a state of defocused attention is reflected in brain activity patterns in highly creative individuals.^87^^,^^88^^,^^89^^,^^90^^,^^91^ They observed lower cortical activation in the frontal lobe. This activation suggests a loose associational thinking style that engages more remote associates.^87^^,^^88^^,^^89^^,^^90^^,^^91^ Nevertheless, evidence that highly creative individuals exhibit lower traits or basal cortical activation remains equivocal.^89^^,^^92^ Similar EEG patterns in frontal brain regions were observed during creative divergent thinking and mental relaxation.^92^ These findings contrast with those identified during creative convergent thinking. Alpha waves, which fall within the 8–12 Hz frequency band, are sensitive to higher-order cognitive abilities.^93^^,^^94^^,^^95^^,^^96^^,^^97^ Semantic memory influences attentional processes, as indicated by decreases in alpha band power.^97^ In contrast, Pfurtscheller and Lopes da Silva^98^ propose that an increase in event-related alpha power signifies a state of cortical inhibition. During creative idea generation, especially in divergent thinking-related tasks, alpha power is modulated.^28^^,^^99^^,^^100^^,^^101^^,^^102^^,^^103^^,^^104^ Increased alpha power in the right parietal cortex during CT suggests the existence of a brain-gating mechanism. This mechanism allows individuals to focus on internal information while minimizing distractions from external stimuli.^104^ Thus, the modulation of alpha oscillations serves as a biomarker. It efficiently eliminates external distractions and promotes internally directed attention, both of which are crucial for effective CT.^104^^,^^105^^,^^106^

In real-world situations, the activation of selective mechanisms enables access to relevant information stored in long-term memory, as outlined in the Attention-to-Memory (AtoM) model.^107^ This process also involves integrating episodic and semantic prior knowledge. The AtoM^107^ offers a framework for explaining how attention mechanisms selectively shape memory encoding and retrieval. Retrieval is attention-dependent, requiring focused or diffuse attention to access relevant memories.^72^ AtoM suggests that top-down attention enriches information encoding by selectively focusing on relevant stimuli. Conversely, bottom-up attention involuntarily leads to the presentation of less relevant information. This duality controls CT through the interplay between WM and AC.^8^^,^^12^^,^^108^^,^^109^ WM requires focus on key information while retrieving various associations, and AC organizes how memories are stored and recalled. As such, CT emerges from AC to rule dynamic access, recombination, and iterative refinement of stored information. In attention research, scholars differentiate between two functional networks: the ventral attentional network (VAN) and the dorsal attentional network (DAN).^110^^,^^111^^,^^112^ VAN is involved primarily in stimulus-driven reorienting, while DAN mainly controls voluntary attention. The temporo-parietal junction (TPJ) is a crucial part of the VAN.^113^ Berkowitz and colleagues observed diminished activation in the right TPJ in classically trained musicians relative to non-musicians during melodic improvisation.^113^ This finding emphasizes the functional role of the right TPJ in VAN; when deactivated, it impairs the ability to redirect AC toward task-irrelevant stimuli. This deactivation, commonly observed in musicians during improvisation, may signal increased reliance on top-down processing.^113^ As a result, trained musicians might hold back responses to stimuli related to their playing while they plan their following improvised sequences.^113^ This ability to filter out external distractions enables individuals to focus on generating internal thoughts without being distracted by external stimuli.

The relative contributions of the VAN and DAN control systems are associated with states of CT that may be induced either spontaneously or deliberately.^114^ By synergistically combining these two attentional pathways, we can improve the efficacy of CT.^115^ The optimal sequence for maximizing the usefulness and novelty of creative outputs includes periods of goal-directed attention followed by periods of undirected attention.^115^ Within this framework, the DAN pathway posits that a directed, goal-oriented form of attention increases deliberate information processing, thereby enabling the development of creative ideas.^115^ The VAN pathway, an undirected, broad form of attention, fosters spontaneous, associative processing, thereby generating novel ideas. Yeo’s perspective^115^ on the contribution of AC to CT does not merely imply engagement of the fronto-parietal (FP) attention networks. Instead it emphasizes the functional coupling of these AC networks with other large-scale neural systems involved in CT.^109^^,^^115^^,^^116^^,^^117^^,^^118^^,^^119^ Specifically, the dorsal and ventral regions of the FP attention networks contribute to CT by filtering external information—potentially interfering with it—and by voluntarily focusing on internal thoughts, thereby generating novel creative ideas.^120^

Further empirical evidence indicates that during idea-generation tasks, the interaction between internally focused attention and memory retrieval processes activates extensive brain networks.^73^^,^^74^ These networks support controlled semantic (meaning-based) searches. They also support flexible integration of episodic (experience-based) details. Creativity-based semantic associations define CT as the activation of remote ideas and concepts.^14^^,^^86^^,^^121^^,^^122^^,^^123^^,^^124^ Indeed, attention-based explanations conceptualize CT as the ability to activate ideas and concepts that are remotely represented and not immediately accessible or obvious. Engaging in this process requires the deployment of cognitive resources and involves the brain’s mechanisms for encoding relevant information to generate novel ideas.^74^^,^^86^^,^^125^^,^^126^ Recent research elucidated the significance of a conceptual knowledge base for CT by delineating unique temporal and prefrontal contributions via a lesion methodology (studying individuals with brain injuries).^127^^,^^128^^,^^129^ Moreover, the engagement of the semantic system—including left-lateralized fronto-temporal-parietal regions—has been consistently reported in functional imaging studies^130^^,^^131^; see also Cogdell-Brooke et al.^132^ for a meta-analysis.

The prominent neural networks for CT encompass the DMN, the salience network (SN), and the executive central network (ECN); for review, see Khalil and Demarin.^37^ DMN involves TPJ, which is part of the VAN, and is activated at rest and during self-referential processing.^133^^,^^134^ Several functional roles in CT are attributed to the DMN. These include associative processes and remote associative thinking.^135^^,^^136^ The DMN facilitates idea generation by integrating various representations. Emerging evidence highlights its role in idea assessment, suggesting that it generates ideas and evaluates candidate ideas based on memory and originality. In contrast, the SN—consisting of the insula and dorsomedial PFC—is involved in selective processing.^137^ Patil and colleagues identified shifts in connectivity dynamics among the SN, DMN, and dorsal FP network during the remote associate task.^138^ ECN, which includes the lateral part of the PFC, contributes to the effective allocation of WM resources, allowing focus on relevant information and suppressing distractions during the phase of idea generation.^8^^,^^26^^,^^115^^,^^119^^,^^139^ Therefore, the interplay between sustained AC and robust WM capacity supports ECN's dynamic engagement in CT. This engagement fosters both controlled and spontaneous modes of thought, enabling the generation of original ideas.^28^ Taken together, AC networks operate collaboratively rather than in isolation. They engage with other extensive neural networks, particularly the DMN, SN, and ECN, to direct (or redirect) processing resources toward relevant representations during CT.

## Creative therapy and attention-deficit/hyperactivity disorder

The necessity of creativity, as recognized in the hierarchy-of-needs theory, is integral to the human need for self-actualization—the intrinsic motivation to realize one’s unique potential.^140^ Biases in information-processing mechanisms, such as cognitive disinhibition, may alter the tendency toward increased creativity and raise the risk of psychopathological features.^141^^,^^142^^,^^143^ This connection emphasizes shared characteristics, including a high tolerance for ambiguity. Creative professions often involve a high degree of uncertainty and associated insecurities at many levels.^144^ This aspect ranges from the lack of guarantees in individual creative endeavors to ongoing job instability and the rarity of prolonged success. Such factors can initiate considerable psychosocial stress, which may ultimately result in deteriorating mental health. While substantial scientific research, particularly in psychology, indicates a moderate and complex relationship between creativity and mental illness,^145^^,^^146^ there is growing evidence of an opposing trend in public or community mental health contexts. This contrasting trend supports the use of creativity within the framework of “art therapy/art therapies” or “creative art therapy/therapies” to address a variety of health-related disorders.^38^ The rationale is that engaging in creative activities may improve mental well-being.^37^^,^^147^^,^^148^ While it may seem paradoxical for both concepts to coexist, they can be compatible, as they represent different aspects of the same associative connection,^149^ and denote distinctive degrees of functioning.

In the context of CATs, we should acknowledge that factors beyond creativity alone may contribute to improvements in well-being and in health-related disorders associated with participation in various art forms. These factors encompass motivation, social interaction, and emotional engagement.^39^ Therefore, for individuals with ADHD, the advantages of CATs may stem from the therapeutic interactions and supportive environments rather than from the creative process itself. Nevertheless, activities that foster engagement and social interaction can develop a sense of structure, allowing ADHD individuals to explore their creative potential more freely. In this context, a flexible attention strategy may help them refocus. We discuss how the application of CATs through engagement in activities such as art, music, dance, drama, and writing, hold promise by coupling the brain’s plasticity.^37^^,^^150^^,^^151^ Engaging in such activities promotes structural and functional plasticity, thereby strengthening various neural networks associated with AC.

Consequently, CATs may provide substantial benefits for individuals with ADHD.^152^^,^^153^ Persistent patterns of deficits in AC, impulsivity, and hyperactivity are characteristic features of ADHD.^154^ These deficits significantly impact multiple aspects of functioning—AC^155^^,^^156^ (mostly selective and sustained attention^157^), inhibition,^158^ CF,^159^ WM,^160^^,^^161^ temporal information processing,^162^ emotional regulation,^156^^,^^163^ and motor skills.^164^^,^^165^ Thus, using creative activities as therapy might moderate key deficits in AC, thereby improving the quality of life for individuals with these conditions. Therapeutic approaches have shown promise in alleviating attention problems in children with ADHD. These interventions commonly include training to strengthen AC, impulse control, and emotional regulation.^166^^,^^167^^,^^168^ Thirty-five years ago, a study assessed the creative aptitudes of children with ADHD and found that these individuals exhibited higher figural creativity than matched controls.^169^ However, two follow-up studies found no significant correlation between ADHD and CT, nor did they provide evidence to disprove it.^170^^,^^171^ In contrast, two subsequent studies established a link between ADHD and CT.^172^^,^^173^ Further studies have indicated that individuals with ADHD exhibit greater creative accomplishments and self-reported creative behaviors than their peers.^174^^,^^175^^,^^176^

Despite this promising outlook, the precise correlations and causal mechanisms by which CATs influence attention-related disorders remain unclear and could be elucidated through rigorous experimental methodologies. Employing such methodologies in upcoming research on the effectiveness of CT across domains, such as verbal and figural, is necessary to resolve conflicting findings. We can approach CATs from multiple perspectives, with brain plasticity presenting a notably compelling viewpoint.^37^^,^^150^^,^^151^ While there is evidence indicating that CATs may be beneficial for ADHD, there are several limitations. The first limitation is the lack of sufficient empirical data, which calls for systematic, robust, and rigorous studies to validate these interventions and to address heterogeneity regarding current conclusions. Furthermore, most creativity studies tend to focus on a single age group—primarily student samples—or rely on qualitative reports, which limits the ability to draw definitive conclusions about the explicit benefits for individuals with ADHD. Moreover, most research on neuroplasticity stems primarily from studies on generalized cognitive training. Given these circumstances, we should carefully evaluate claims about the neuroplasticity associated with CATs. We should refrain from making definitive assertions until we can provide direct evidence to support these claims.

## Challenges and recommendations

We outline the constraints on the effective use of CATs for ADHD and provide suggestions to mitigate them. CT involves shifting attentional focus.^22^^,^^26^^,^^32^ Defocused attention enables a thorough evaluation of ideas, leading to novel connections.^8^^,^^24^^,^^139^ However, most CT studies examine executive functions in isolation and do not adequately provide a thorough analysis of AC's role across the various phases of CT. Neuropsychological studies often isolate each phase or only address transitions. The lack of co-registration across neurophysiological measures adds further challenges to interpreting experimental data. Another issue is the lack of diversity in study samples: most creativity research relies on homogeneous participant groups and often neglects demographic factors such as age, ethnicity, and clinical history. These limitations construct barriers to creating a complete picture of the interactions among various neural processes during CT-related tasks. Thus, the precise role of AC in verifying the originality of ideas during the final stages of CT, as well as its potential function in earlier stages, remains elusive (Box 1). Moreover, the role of AC at each CT stage (Figure 1A) and the extent to which these stages differ across creativity domains (Figure 1B) remain unclear. A primary reason for this elusiveness is that the existing static assessments of CT do not capture its dynamic essence during the phases of idea generation and evaluation. In a similar vein, the current predominance of these tasks may not capture the full range of creative mental operations.^177^ Providing computational predictions of these dynamics presents a promising approach to address this challenge. By analyzing the processes involved in idea generation, this prediction assesses how creative ideas are identified. These processes could involve the computation and prediction of the associated learning rules proposed by Khalil and Moustafa.^178^

Building on these theoretical and methodological challenges, most research on creativity is conducted in laboratory settings. This approach often neglects the influence of environmental context and the real-world applications of the creative process. Furthermore, creativity in humans transcends mere segregation of executive functions; it also requires evaluating emotional and social contexts.^10^^,^^37^ These limitations diminish the relevance of research findings to real-world creative tasks and problem-solving situations outside laboratory settings. To bridge this gap, designing experimental contexts that more closely mirror our natural environments could reflect how these systems interact dynamically during CT and its associated phases. Therefore, we call for studies that assess creativity in naturalistic settings (e.g., workplaces, art studios, educational environments). An elucidation of the interaction between selective attention processing and access to internal knowledge—particularly semantics and episodic memory—can benefit research on CT in real-world contexts. This approach would allow us to explore how a wider array of emotional states, extending beyond basic emotions such as calmness and excitement, can influence CT-based AC. This investigation could employ various psychophysiological measures and mobile brain imaging techniques. Implementing this experimental design would therefore uncover intricate mechanisms across various phases and facilitate analysis of variations in AC dynamics in real-world contexts.

In addition to context-related gaps, another challenge is the lack of longitudinal studies on the impact of AC on CT development. In 1940, Lewis Terman initiated a groundbreaking longitudinal study.^179^ His extensive research exemplifies pioneering work in creativity research, as Terman identified 1,528 gifted pupils and tracked their progression into adulthood to determine how many of them made significant creative contributions.^179^ Ultimately, he found that none did. The data indicate that intelligence influences creativity only up to a certain threshold; beyond that, there is no significant correlation between intelligence and creative achievement The data analysis reveals that adult success is largely determined by social adjustment, emotional stability, and motivation once an IQ (IQ is a score that indicates an individual's ability to think, solve problems, and comprehend concepts relative to their peers of the same age.) of 140 is attained.^179^ Accordingly, longitudinal studies could facilitate monitoring shifts in CT and AC across developmental phases while incorporating educational or artistic training. Moreover, future experiments should encompass a broader range of demographics, including age, culture, and cognitive styles, to establish a standard database for analyzing individual differences in creative potential and associated cognitive processes. The databases should also include a diverse array of creativity tasks spanning domains such as music, art, science, movement, and writing. The availability of this comprehensive database enables researchers to access a larger sample size, thereby improving the generalizability of current research findings across diverse populations.

**Figure 2:**
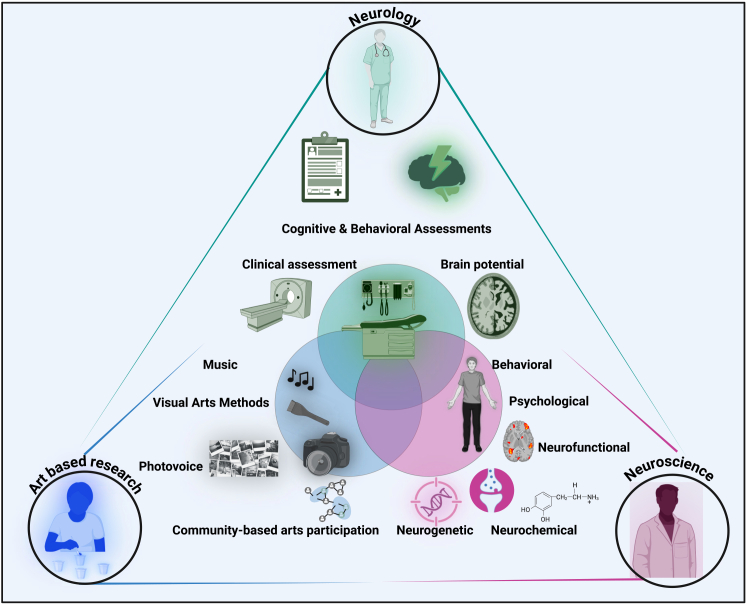
Structuring the interdisciplinary research agenda This figure depicts the diversity of research by incorporating interdisciplinary approaches across neurology, neuroscience, and art-based research. Neurology emphasizes brain potential, cognitive and behavioral assessments, and clinical evaluations. Neuroscience employs a range of approaches, including behavioral, psychological, neurofunctional, neurochemical, and neurogenetic methods. Art-based research relies on community-based art participation, photovoice, visual art, and music, as well as other art forms such as dance and drama. The integration of diverse metrics from each discipline improves the quality of evaluation methods used in the current research.

Addressing diverse methodological and conceptual challenges requires targeted action. We recommend promoting dialogue across disciplines and fostering collaboration within and between them. For instance, a recent study linking creative experience to brain clock exemplifies this dialogue.^180^ Thus, we call for the establishment of a comprehensive, interdisciplinary research agenda that unites the expertise of neuroscientists, art therapists, clinicians, and other professionals.^37^^,^^39^^,^^181^ This database should compile empirical findings from multiple disciplines utilizing robust interdisciplinary methodologies (Figure 2).

Future research should involve experts from basic science, neurology, and psychiatry, which is fundamental to structuring an interdisciplinary agenda (Figure 2). This agenda facilitates comprehensive explanations of the temporal dynamics of neural processing, which, in turn, allow for a thorough evaluation of the impact of CT on ADHD. The explanations would clarify the mechanisms that underlie CATs. We propose that neuroscience should take the lead in fostering an interdisciplinary approach that incorporates psychological and computational methods. Thus, collaboration among neuroscientists, psychologists, and computational modelers can address gaps in both empirical and theoretical research regarding CT-based AC. This approach can elucidate how CATs effectively address ADHD. For example, it enables analysis at the microscopic, mesoscopic, and macroscopic levels across neural, behavioral, and emotional systems. This analysis can clarify how optimizing neural signals may improve the prediction of AC-related CT and its relationship to the development of ADHD. Methods such as distributed signal processing, parallel computation, and information integration could be beneficial in this context.

Aesthetic psychology supporting detailed assessments of artworks and the experience of viewing or creating them, especially in relation to health and neurodegenerative diseases.^182^^,^^183^^,^^184^^,^^185^ In this setting, we can analyze a visual artwork by identifying its attributes. By combining these sensory features with their respective weights, we can determine a subjective aesthetic value for the stimulus. Different individuals may assign dissimilar weights to feature integration, underscoring their variability in sensory processing. Creativity research on animal models could benefit from interdisciplinary approaches; however, several questions remain unanswered (Box 1). Moreover, there is a difference between creative/innovative behaviors in humans and rodents. Human creativity encompasses recombinative innovation, wherein pre-existing concepts are integrated into novel configurations of ideas or products. In contrast, the creative/innovative behaviors observed in rodents primarily focus on behavioral adaptation rather than the creation of original ideas. Therefore, the creative/innovative behaviors exhibited by rodents may lack the hierarchical organization of concepts essential to higher-order creative processes, limiting their comparability with human creativity. Nevertheless, this limitation may not apply to non-human primates. For instance, non-human primates exhibit a closer resemblance to human cognitive abilities^186^. Although differences in neural architecture and variations in structural and functional connectivity among species may restrict the direct application to human creativity, they still provide a meaningful characterization of neural circuits that we currently lack. Another limitation that we face is that creativity research on cognitive training frequently emphasizes three neural networks—specifically the DMN, SN, and ECN^37^ —while neglecting other crucial brain regions, such as the cerebellum,^178^ resulting in an inadequate identification of the neural foundations of CT. A potential strategy to address this limitation could involve correlating neuroimaging findings associated with creativity and brain disorders with shared brain circuits.^187^ We should adopt this approach while simultaneously collecting new data to establish a comprehensive mapping of CT-based AC that goes beyond the existing neural network models. In a similar vein, we should thoroughly evaluate CT-based AC that accounts for emotional factors to clarify how emotions influence the development of CT in individuals with ADHD by monitoring changes in their sensory information processing over time.

**Figure 3:**
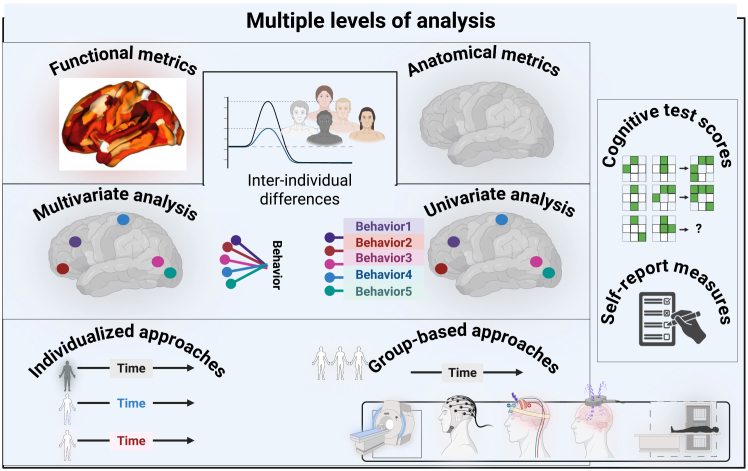
Diverse research methodologies and multiple analytical levels This graph presents functional measures of brain activity. It also includes statistical analyses that consider multiple variables simultaneously, known as multivariate analyses, as well as approaches tailored to individual subjects. These methods build on earlier techniques that relied solely on anatomical matrices or single measurements, known as univariate methods. Both tailored and group-based approaches combine multiple brain-scanning methods. fMRI measures brain activity by detecting changes in blood flow. EEG records electrical signals from the scalp. These are used alongside brain stimulation techniques. These include noninvasive methods such as tDCS, which uses weak electrical currents on the scalp. Invasive techniques such as TMS use magnetic fields to stimulate neurons. Often, these techniques are used together. PET is also used; it measures dopamine activity by tracking a radioactive tracer in the brain, complementing the other methods. The acronyms used here are fMRI (functional magnetic resonance imaging), EEG (electroencephalography), tDCS (transcranial direct current stimulation), TMS (transcranial magnetic stimulation), and PET (positron emission tomography).

Theories of grounded and embodied cognition propose that recalling past stimuli engages a multimodal process^188^^,^^189^ that triggers the simulation of sensory inputs associated with those prior experiences. A comprehensive evaluation of ADHD requires employing a multi-level analysis alongside a multiple-case study design. The implementation of these experimental designs allows for the application of various methodologies to assess changes in CT and their relationship with AC in both nonclinical and clinical populations, particularly among individuals diagnosed with ADHD. Interdisciplinary collaboration (Figure 2) supports the integration of diverse experimental levels and their associated analyses, thereby providing pragmatic solutions to these challenges. The experimental variations include functional brain matrices, multivariate analysis, and personalized methods that reflect individual differences and cognitive test outcomes (Figure 3).

We should leverage the advantages of multi-level analyses and build upon previous studies that utilize anatomical matrices, univariate analyses, group-based methodologies, and self-reported measures (Figure 3). These analyses can formulate new assessments that combine individualized and group-based neuroimaging techniques, such as functional magnetic resonance imaging (fMRI), electroencephalography (EEG), and various brain stimulation techniques. Employing time-dependent methodologies facilitates the investigation of the interaction between focused and diffuse attention during the phases of CT. Combining fMRI and EEG, or employing diffusion fMRI, enables analysis of real-time brain activity and underlying neural connectivity during creative tasks. This approach can reveal dynamic patterns in brain processes linked to neural mechanisms of AC associated with CT. A recent study by an interdisciplinary team used integrative neuromethods, specifically fMRI and EEG, to explain how creative experiences across diverse domains can influence the brain’s clock.^180^ Brain stimulation is another neuromethod, encompassing both noninvasive procedures, such as transcranial direct current stimulation, and invasive ones, such as transcranial magnetic stimulation. Positron emission tomography is frequently used to assess biological neuromodulation, particularly dopamine activity. Finally, using machine learning techniques to improve precision in analyzing brain imaging data and predict CT based on individual differences in structural and functional neural connectivity could open new therapeutic avenues for ADHD.

## Discussion

**Figure 4:**
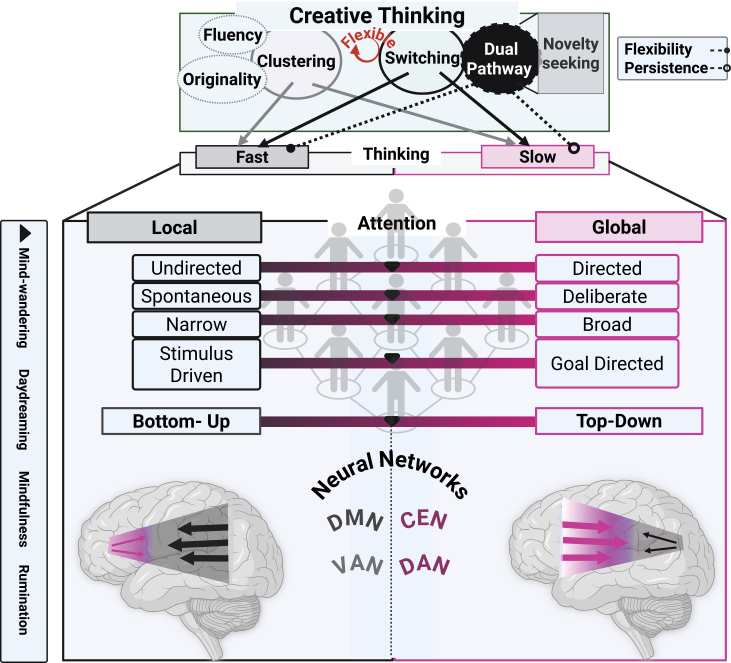
A holistic framework for creative thinking based on attention control This framework is partially adapted from Khalil and Brüne.^13^ It comprises two systems—clustering and switching—that function through temporal modulation, affecting both fast and slow thinking while accounting for individual differences. Clustering, associated with fluency and originality, operates alongside switching, which engages the creativity pathways of flexibility. In this context, persistence is characterized by slower transitions, while rapid transitions indicate flexibility. The interplay between bottom-up and top-down processes reinforces this duality and corresponds with the dual systems of local and global attention. These systems, in turn, highlight the trade-offs between parallel modes: undirected and directed, spontaneous and deliberate, narrow and broad cognitive modes, and stimulus-driven and goal-directed approaches. Here, the extent of interaction between bottom-up and top-down mechanisms fosters dynamic competition among the DMN, VAN, CEN, and DAN, which underscores individual variability in these trade-offs. Overall, this dynamic signature may elucidate how the brain adjusts its resource allocation in response to environmental demands, as reflected in behaviors such as mind-wandering, daydreaming, mindfulness, and rumination. The acronyms of this illustration are DMN, the default mode network; VAN, the ventral attention network; CEN, the central executive network; and DAN, the dorsal attention network.

We outline creative mental operations grounded in attentional processing (Figure 1) . We provide an overview of how CT-based AC operates at phenomenological and neurophysiological levels. CT functions within the AC^8^^,^^18^^,^^24^^,^^32^ to encompass conceptual knowledge based on memory systems.^8^^,^^12^^,^^74^^,^^83^^,^^86^^,^^125^ These memory systems (semantic and episodic) serve as the foundation for accessing, retrieving, and recombining information to generate novel ideas/solutions.^14^^,^^86^^,^^121^^,^^122^^,^^125^^,^^128^^,^^129^^,^^130^ The dynamic interplay among DAN/VAN, the DMN, the ECN, and the SN may explain the process of CT.^109^^,^^116^^,^^117^^,^^118^ Nevertheless, debates continue regarding the precise mechanistic mapping of AC and its associated systems in CT. Evidence from a recent multicenter study by Chen et al.^119^ suggests that CT is characterized by flexible transitions between the DMN and ECN brain networksThis flexibility facilitates both spontaneous and controlled cognitive processes. We propose an overarching framework based on AC's duality to captures the fluidity of cognitive processes across CT’s phases. This framework also clarifies their dynamic relationship (Figure 4).

Our framework focuses on individual differences and how two dual processes—clustering and switching—are temporally modulated (Figure 4). Clustering is associated with fluency and originality, and switching is linked to flexibility and persistence (for a review, see Khalil and Brüne^13^). This duality is also evident in the pathways of switching: a flexible pathway allows for rapid switching, while a persistent pathway supports gradual switching. Clustering and switching also operate within other dual systems. These systems combine local and global attention mechanisms and bottom-up and top-down processes. This duality exemplifies a dynamic trade-off among diverse cognitive processes: undirected vs. directed, spontaneous vs. deliberate, narrow vs. broad, and stimulus-driven vs. goal-driven (Figure 4). Variations in these trade-offs among individuals shape the dynamic interactions and competition among DMN, VAN, CEN, and DAN. These differences modulate brain states such as mind-wandering and daydreaming, mindfulness, and rumination. Mapping such dynamic processes that drive dual systems may explain how the brain adapts resource allocation, responds to environmental demands, and achieves a balance of adaptability and efficiency in CT. Thus, our framework may offer a theoretical basis for forthcoming research endeavors to explain CT dynamics through AC, beyond neural circuits.

Implementing this framework in empirical research requires careful consideration of methodological limitations, sample diversity, and variations in experimental task design, as discussed in the challenges and recommendations section. Progressing in therapeutic directions necessitates integrating theoretical and empirical research agendas across disciplines. Our primary recommendation is to structure the interdisciplinary research agenda by incorporating neuroscience, neurology, and CATs and drawing on expertise from emotion, computation, and psychiatry (Figure 2). Establishing such an agenda facilitates mapping CT and AC-related mental processes at behavioral and neural levels using multi-level approaches (Figure 3). Prioritizing a robust, interdisciplinary plan is key to advancing the effectiveness of the practical application of CATs. Creative activities—such as art, music, drama, and dance—may improve cognitive, emotional, and social functioning in individuals.^190^^,^^191^ Engaging in these activities may benefit individuals with ADHD; however, they might not be effective for everyone. Some individuals may need alternative therapeutic approaches. This is why we emphasize individual differences. Recent research suggests creative experiences augment neuroplasticity and may decelerate brain aging.^180^ Accordingly, engaging individuals in meaningful and expressive activities offers alternatives to conventional therapies. By grounding the creative process in AC and individual differences (Figure 4), we could set new guidelines and open clear therapeutic avenues, especially for those with ADHD.

## Acknowledgments

S.B.S.N. acknowledges support from the SAFAR doctoral scholarship (111751P, 129650N, and 151556P), funded by the Embassy of France in Lebanon and the Conseil Régional de Nouvelle Aquitaine (AAPR2021A-2020-12051410). E.M. acknowledges support from a senior chair in fundamental research at the Institut Universitaire de France. R.K. acknowledges support from the Constructor University's DEAL agreement.

## Author contributions

Conceptualization, investigation, and writing – original draft, S.B.S.N.; conceptualization, investigation, funding acquisition, and writing – original draft, E.M.; conceptualization, investigation, writing – original draft, and writing – review and editing; conceptualization, visualization, investigation, supervision, writing – original draft, and writing – review and editing, R.K. All authors gave final approval for publication and agreed to be held accountable for the work presented here.

## Declaration of interests

The authors declare no competing interests.

## Supplemental information

Video S1. Attention unleashed: Video recap
